# The Hospital Sunday Fund and the "Medical Press"

**Published:** 1905-01-07

**Authors:** 


### THE HOSPITAL SUNDAY FUND AND THE "MEDICAL PRESS."

THE *Medical Press* is silent in its issue of the 4th instant in regard to the error into which it fell as to the constitution of the Hospital Sunday Fund, to which we called attention last week. The *Medical Press*, however, returns to its first love, and publishes a long article dealing with the sale of the site of the Royal Orthopædic Hospital. The writer still labours to show, despite the letter his journal has received from the solicitor of the Sunday Fund, and the statement of the secretary of the Royal Orthopædic Hospital, that "the Metropolitan Hospital Sunday Fund took no part whatever in advising the hospital to dispose of its site, or in offering any opinion as to its value," that the Sunday Fund did interfere ; and, had it had its way, the Royal Orthopædic Hospital would have lost £12,000. We would invite our readers to peruse the article in the *Medical Press* in order to realise the weakness of the case put forward, and to confirm their confidence in the administration of the Hospital Sunday Fund. In effect the only new point made is that the *Medical Press* "holds the definite assurance of a member of the deputation, consisting of the honorary solicitor, one of the surgeons, and the secretary, that the chairman of the Fund said in so many words that the hospital should be sold." On this point Sir Edmund Hay Currie will be able to speak with authority, and, should this point be disposed of, we have no doubt that the misapprehension of the *Medical Press* will be removed, when it will promptly withdraw its charges and so end the matter. Meanwhile we may remind the *Medical Press* that it might be well to look up the discussions which took place at the meetings of the Governors of the Royal Orthopædic Hospital, as they reveal the weakness of the opposition in point of numbers and otherwise.

